# Impaired Innate Immunity Mechanisms in the Brain of Alzheimer’s Disease

**DOI:** 10.3390/ijms21031126

**Published:** 2020-02-08

**Authors:** Martina Romagnoli, Elisa Porcellini, Ilaria Carbone, Robert Veerhuis, Federico Licastro

**Affiliations:** 1 Department of Experimental, Diagnostic and Specialty Medicine, School of Medicine, University of Bologna, Via S. Giacomo 14, 40126 Bologna, Italy; martina.romagnoli87@gmail.com (M.R.); ilaria.carbone2@unibo.it (I.C.); federico.licastro@unibo.it (F.L.); 2 Department of Clinical Chemistry, Vrije Universiteit Amsterdam, Amsterdam UMC, 1105 Amsterdam, The Netherlands; r.veerhuis@amsterdamumc.nl; 3 Department of Psychiatry, Vrije Universiteit Amsterdam, Amsterdam UMC, 1105 Amsterdam, The Netherlands

**Keywords:** Alzheimer’s disease, viral infection, antiviral genes, gene expression, brain immunity

## Abstract

Among environmental factors likely associated with Alzheimer’s disease (AD), persistent virus infections, and age-related progressive decline of immune competence might play a pivotal role. However, AD antimicrobial brain immune responses are poorly investigated. The present study focused on genes involved in antimicrobial defenses, especially against virus infections, in the AD brain. In particular, mRNA levels of IRF7, MED23, IL28B, and IFN-α genes were analyzed in hippocampus and temporal cortex brain samples from AD and non-demented controls. All subjects were also genotyped for APOE ε, IRF7, MED23, and IL28B gene polymorphisms. Most AD patients showed decreased mRNA levels of all investigated genes in the hippocampus and temporal cortex. However, a small group of AD patients showed increased hippocampal mRNA expression of MED23, IL28B, and IFN-α. mRNA levels of MED23, IL28B, IFN-α from the hippocampus and those of MED23 from the temporal cortex were further decreased in APOE ε4 allele AD carriers. Moreover, rs6598008 polymorphism of IRF7 was significantly associated with decreased hippocampal expression of IRF7, MED23, IL28B, and IFN-α. These findings suggest that AD brains show impaired innate antimicrobial gene expression profiles, and individual genetic makeup, such as positivity for the APOE ε4 and IRF7 A alleles, might affect brain immune efficiency.

## 1. Introduction

Alzheimer’s disease (AD) is a chronic neurodegenerative disease and the most frequent form of dementia in the elderly [1]. It is of interest that declining immunity during aging is often associated with persistent antigen stimulation and peripheral chronic inflammation [2]. Moreover, neuroinflammation has emerged as a relevant component of AD brain pathology [3].

Pathogens, such as viruses of the herpes family, through frequent cycles of reactivation and latency, constantly trigger the immune system, which is not able to completely eradicate these microbes. Therefore, persistent neurotropic pathogens might play a role in microglia activation in the brain of genetically susceptible elderly and contribute to neurodegenerative processes [4,5].

The amyloid-β (Aβ) peptide, which is associated with neurodegenerative processes of AD, showed antimicrobial activity against eight common and clinically relevant microorganisms [6]. Aβ peptide shares several chemical and biological characteristics of antimicrobial peptides (AMPs), which are components of the innate immune system [6]. Moreover, Aβ peptide showed a protective activity against in vitro infection by the neurotropic virus, herpes simplex virus 1 (HSV-1) [7]. This observation reinforces the notion that persistent and latent herpes virus infections may lead to Aβ overproduction that, in turn, contributes to amyloid plaque formation [7].

Recent findings have confirmed a causative role of viruses of the herpes family in AD [8] and the relationship between human herpes virus 6 (HHV-6) and 7 (HHV-7) DNA abundance and expression of amyloid precursor protein modulators along with induction of other several genes previously found associate with AD risk.

We previously suggested that the concomitant presence of a set of single nucleotide polymorphisms (SNPs) associated with AD might result in a genetic signature predisposing to AD via complex and diverse mechanisms, each contributing to modulate individual susceptibility to herpes virus infection [5]. According to this view, efficient immune response is mandatory to preserve brain structure and functioning during aging, and chronic brain infections might play a pathogenic role in the clinical progression of sporadic AD in the elderly with declining immune efficiency [4].

Data regarding the efficiency of human brain immune responses in AD is scanty, and in the present paper, we investigate the brain expression of a selected set of genes belonging to the innate immunity and involved in antivirus defense, such as interferons.

Different members of the Interferon (IFN) family play a pivotal role in the human anti-viral defenses [9,10]. IFNs can be produced by several cell types and primarily act as antiviral and immune-modulatory cytokines [9,10,11]. IFN-α/β or type I IFN and IFN-λ or type III IFN bind distinct receptors. However, they regulate the same sets of genes and show similar biological activities [10,11,12,13].

IFN-λ is a recently discovered group of defensive molecules comprising several members such as IFN-λ1, IFN-λ2, and IFN-λ3, also known as IL29, IL28A, and IL28B, respectively [13,14]. An IFN-λ4 type has also been described [15,16]. Up-regulation of IFN-λ transcription has been shown to depend on the same stimuli, sensors, and signal transduction pathways, as those involved in type I IFN production [11].

In the mouse brain, IFN-α/β was readily produced after infection with various viral RNAs, whereas IFN-λ expression remained lower [17].

IFNs expression is regulated by a family of transcription factors named interferon regulatory factors (IRFs), and they show relevant roles in brain antiviral defenses [18]. In fact, IRF7 and IRF9 share different functions, including host defense, cell growth, apoptosis, and immune cell differentiation [19].

Mediator complex (MED) 23 is an anti-viral component of the mediator complex and regulates the transcription of nearly all RNA polymerase II-dependent genes, which in turn up-regulates IFN-λ by interacting with IRF7 [20].

IRF7 was originally identified in the context of Epstein-Barr virus (EBV) infection and has since emerged as the crucial regulator of type I IFNs after activation by pathogen recognition receptors [21].

Type I IFN induction is mainly regulated by IRF3 and IRF7. IRF3 is expressed constitutively, whereas IRF7 is an IFN-stimulated gene, often induced during late phases of virus infection. Therefore, the IFNs induced IRF7 response and IRF7 activation by viral infection provide a positive feedback for further IFNs production. Moreover, both IRF3 and IRF7 regulated the IFN-λ1 gene expression, whereas IRF7 has been shown the major regulator of IFN-λ2/3 genes [22].

Our recent findings showed that SNPs in genes regulating antiviral responses are differentially distributed in AD and influence a differential blood positivity to EBV and HHV-6 in the elderly [23]. Moreover, risk alleles were increased in the elderly progressing to AD [23]. These findings suggested that individual genetic background might play a role in the progression of cognitive impairment by influencing the efficiency of immune responses to persistent viruses.

Knowledge regarding the efficiency of human brain immune responses against bacteria and viruses during aging and AD is limited.

In the present study, the mRNA expression of genes involved in antimicrobial responses, such as IRF7, MED23, IL28B or IFN-λ3, and IFN-α, in hippocampus and temporal cortex specimens from human controls and AD patients was studied. Moreover, the influence of apolipoprotein (APOE) ε 4, IRF7, MED23, and IL28B gene polymorphisms upon mRNA levels of IRF7, MED23, IL28B, and IFN-α genes was also analyzed.

Our findings showed that impaired mRNA levels of IRF7, MED23, IL28B, and IFN-α were present in AD hippocampus and temporal cortex samples. APOE ε4 and IRF7 A alleles negatively affected mRNA levels in AD hippocampus.

Antimicrobial defense mechanisms of innate immunity appear to be impaired in the AD brain, and such alterations might contribute to neurodegeneration.

## 2. Results

Demographic and clinical features, such as age, gender, clinical diagnosis, disease duration (DD), post-mortem interval (PMI), brain weight, cause of death, and brain area, of patients with AD have been reported in Table 1. AD neuropathological diagnosis, Braak and Thal amyloid deposition scores have been summarized in Table 2.

**Figure 1:**
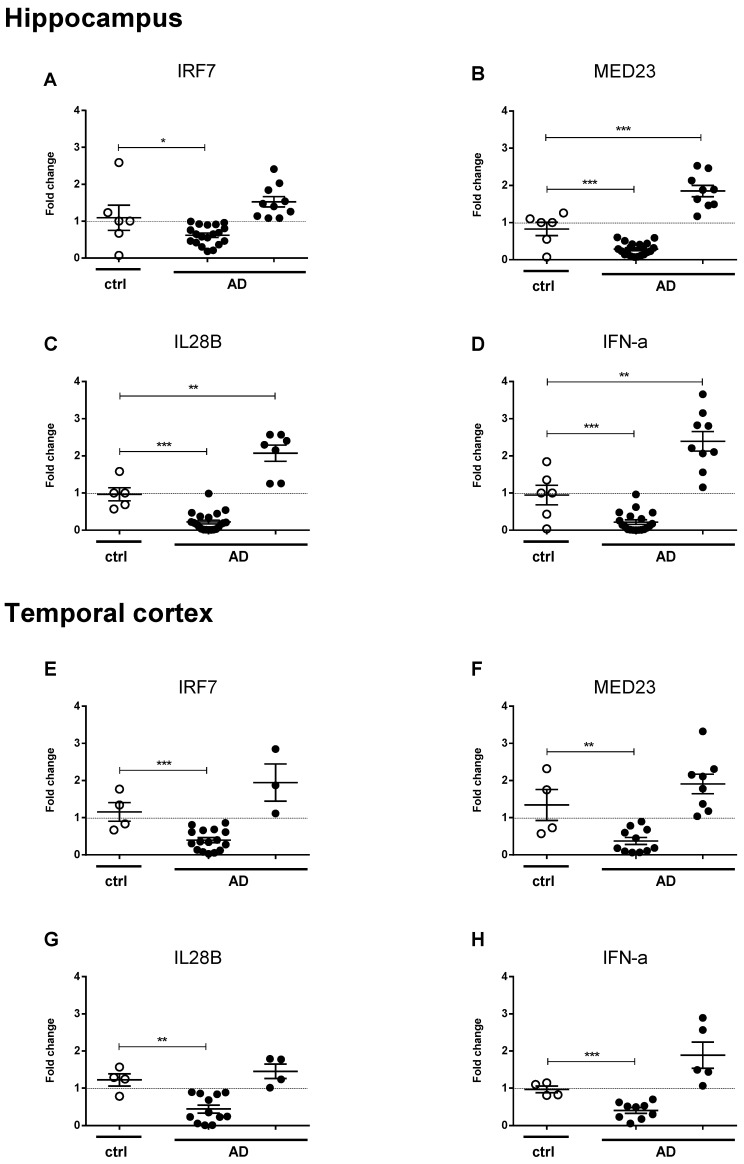
Hippocampal and temporal cortex differential expression of antiviral response genes in Alzheimer’s disease (AD) patients and control subjects. Fold change (q-PCR 2^-∆∆Ct^ method) of IRF7 (**A**,**E**), MED23 (**B**,**F**), IL28B (**C**,**G**), IFN-α (**D**,**H**) normalized to two reference genes (CYC1 and EIF4A2) and relative to control healthy subjects (ctrl), in hippocampus (**A**–**D**) and temporal cortex (**E**–**H**) of patients with clinical and neurological defined diagnosis of AD. Values are given as fold change ± SEM. *p < 0.05; **p < 0.01; ***p < 0.001 (unpaired *t*-test).

Levels of IRF7, MED23, IL28B, and IFN-α mRNA in AD brain hippocampus and temporal samples are reported in Figure 1. The majority of AD brains showed decreased hippocampus mRNA levels of IRF7 (n = 28), MED23 (n = 20), IL28B (n = 21), and IFN-α (n = 19), whilst a small AD group had increased mRNA levels of MED23 (n = 11), IL28B (n = 12), and IFN-α (n = 9).

A similar mRNA expression pattern was detected in AD temporal cortex samples, since a larger AD group showed downregulation of IRF7, MED23, IL28B, and IFN-α genes (Figure 1). A minority of AD showed normal or slightly increased mRNA levels of the above immune factors (Figure 1).

**Figure 2:**
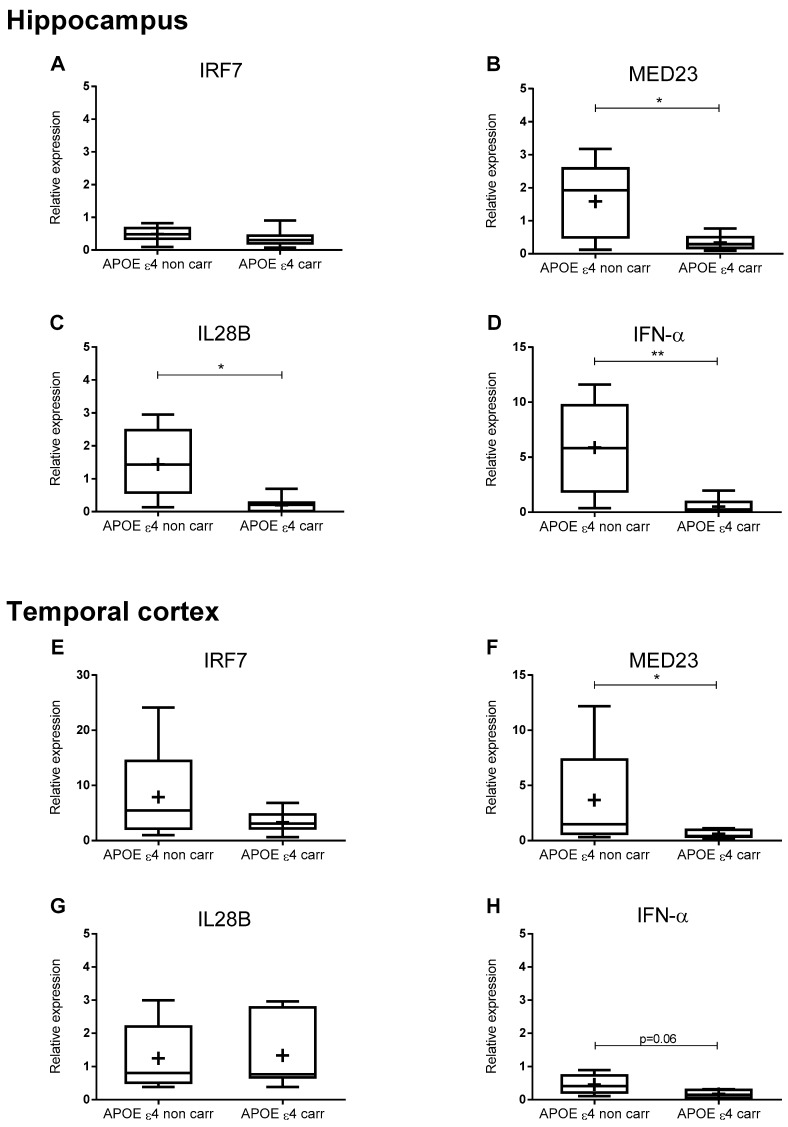
Effect of APOE ε4 allele on antiviral immune gene expression of AD patients. q-PCR data showing relative expression (2^-∆Ct^ values using CYC1 and EIF4A2 as reference genes) of IRF7 (**A**,**E**), MED23 (**B**,**F**), IL28B (**C**,**G**), IFN-α (**D**,**H**) in hippocampus (**A**–**D**) and temporal cortex (**E**–**H**) of AD patients grouped in APOE ε4 noncarrier/or APOE ε4 carrier. Data from each group are shown as a box and whiskers plot with the ends of the whiskers represent the minimum and maximum data values, the horizontal line represents the median, and the “+” represents the mean relative expression values. *p < 0.05 **p < 0.01 (unpaired t-test).

Brain samples were also genotyped for APOE ε polymorphism and gene variations of IRF7, MED23, and IL28B. The presence of APOE ε4 allele was associated with decreased mRNA levels of MED23, IL28B, and IFN-α in AD hippocampus samples and of MED23 in AD temporal cortex samples, as shown in Figure 2.

**Figure 3:**
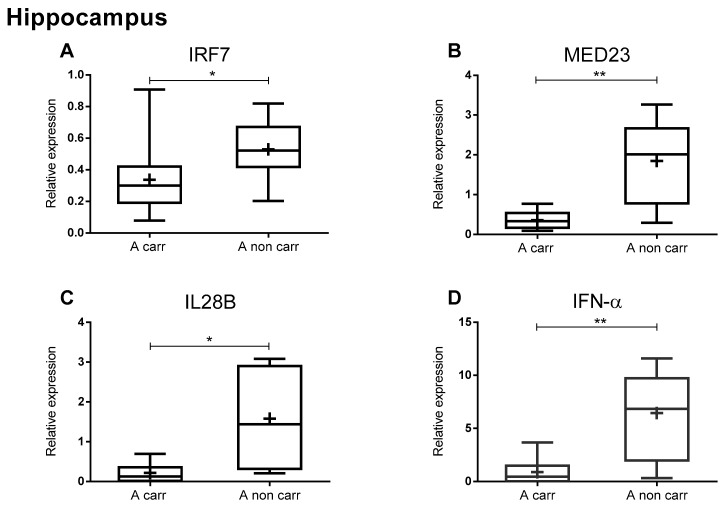
Effect of IRF7 (rs6598008) SNP on antiviral immune gene expression of AD patients. qPCR data showing relative expression (2^-∆Ct^ values using CYC1 and EIF4A2 as reference genes) of IRF7 (**A**), MED23 (**B**), IL28B (**C**), IFN-α (**D**) in the hippocampus of AD patients grouped in A carrier/or A noncarrier. Data from each group are shown as a box and whiskers plot with the ends of the whiskers represent the minimum and maximum data values, the horizontal line represents the median, and the “+” represents the mean relative expression values. *p < 0.05 **p < 0.01 (unpaired *t*-test).

IRF7 gene polymorphism affected IRF7, MED23, IL28B, and IFN-α mRNA levels, and A allele carriers showed significantly decreased levels of the four immune factors in AD hippocampus samples (Figure 3).

No statistically significant difference in mRNA levels of IRF7, MED23, IL28B, and IFN-α from temporal cortex samples between IRF7 A carriers and A noncarriers was observed (data not shown).

The presence of MED23, IL28B, IFN-α gene polymorphisms was not associated with mRNA levels of the four immune factors in both hippocampus and temporal cortex specimens (data not shown).

No relationship between IRF7, MED23, IL28B, and IFN-α mRNA levels and Braak and Braak or Thal scores, duration of the disease, and brain weight was found (data not shown).

## 3. Discussion

The efficiency of immune responses declines with advancing age [24,25], and old age is a major risk factor for AD. However, the role of the immune system in the disease is still unclear. Genome-wide association studies performed in AD reported that several genes with immune regulatory functions were associated with differential risk of the disease [26,27]. However, a more defined role of genes regulating innate immunity with the pathogenesis and clinical history of AD remains to be assessed.

Infections by pathogens, such as the Herpes virus, have been suggested to play a role in the clinical progression of the disease [28]. It is known that even in healthy young persons, the immune system never completely eradicates these pathogens. In the elderly, repeated cycles of activation and latency, along with infective agent persistence, may further impair immune responses and accelerate the senescence of the immune system [2,25]. Moreover, herpes viruses, which are neurotropic, might directly infect and damage selected brain areas in genetically susceptible elderly, contributing to neurodegenerative mechanisms [5], and herpes DNA has been indeed found in AD brains [4,8,25].

Recent findings confirm and extend the association of herpes virus infection with neurodegeneration and AD [8,29,30]. Other pathogens have been implicated in the clinical history of AD [31], and chronic infections are emerging risk factors for the disease [32].

Recent data showed that amyloid-Aβ peptide protected against microbial infection in AD animal models [33]. For instance, Aβ oligomerization was necessary for its antimicrobial activity, and brain infection of 5XFAD mice by *Salmonella Typhimurium* bacterium resulted in an amyloid deposition surrounding the invading bacteria [33], and Aβ peptide has been related to immune defensive mechanisms of the human brain [34]. These data are compatible with the notion that the amyloid-Aβ peptide might be a component of the innate immunity against brain pathogens and virus brain chronic infection may induce its production.

Findings in the present article showed that gene expression of antimicrobial defense factors, such as IRF7, MED23, IL28B, and IFN-α, was impaired in AD brains.

Most AD brains showed decreased mRNA levels of these defensive factors, while a minority of AD patients had increased levels. Differential expression patterns might represent different AD clinical stages. Brains overexpressing the immune factors might actively respond to an active regional infection or tissue insult. After a partially successful immune response, a latent viral phase is induced, and these factors are downregulated. Such a decrement would decrease the concomitant activation of microglia and astrocyte, downregulate neuroinflammation, and mitigate neuronal damages. In other words, we suggest that different expression patterns of immune factors and cytokines may describe different reactivation and latency cycles of infectious agents.

Increased mRNA levels of MED 23, IL28B, and IFN-α were found only in the AD hippocampus, and our data suggest that different brain regions appear to be differentially involved in these chronic inflammatory responses. Replicating neurons in the hippocampus cortex [35] appear to be more susceptible to virus infections or microbial products/toxins. Therefore, persistent inflammation may induce accelerated neurodegenerative mechanisms in this brain area.

Possible regulatory mechanisms of the innate immunity genes upon amyloid-Aβ peptide expression in normal or AD brains have been poorly explored. However, it cannot be excluded that amyloid-Aβ peptide might function as an emergency defensive mechanism and compensate the impaired efficiency of other specialized immune defensive genes in the aging brain.

Here, we showed that MED23, IL28B, and IFN-α mRNA hippocampus levels in AD APOE ε4 carriers were further decreased. The temporal cortex from AD APOE ε4 carriers also showed the lowest values of MED23 and IFN-α mRNA levels.

It is known that APOE affects immunity since increased systemic pro-inflammatory states and altered immune responses have been found in APOE deficient mice [36,37]. Therefore, at least part of the increasing AD risk effect of the APOE ε4 allele might be mediated by a negative influence on the brain’s immune efficiency.

Our data show that IRF7 allele A carrier status was associated with decreased levels of IRF7, MED23, IL28B, and IFN-α expression in the AD hippocampus. These findings reinforce the notion that individual genetic makeup affects brain immune efficiency.

Findings regarding IFN and brain functions are scanty. However, transgenic mice chronically overexpressing astrocyte IFN-α developed a progressive inflammatory encephalopathy and neurodegeneration [38]. Moreover, IRF7 deficient mice produced elevated levels of CXCL13 after virus infection and showed impaired regulation of microglia activity [39].

It is interesting to note that type I-IFNs also play a critical role in regulating tissue homeostasis and regeneration. Insufficient resilience, defined as impaired repair and regeneration of host tissues, rather than inefficient infectious agent clearance, may induce chronic neuroinflammation [40], and inefficient brain resilience over the years might contribute to neurodegeneration in preclinical and clinical AD.

Our results are in accordance with different genetic investigations from genome-wide association studies showing an association of several immune regulatory genes with AD pathogenesis [27]. In fact, subsequent complementary statistics and bio-informatic approaches showed that several single-gene polymorphisms in IFN genes increased AD risk [41]. Results from our investigation further support the relevance of IFN family genes in AD.

Our findings from post-mortem brain samples, however, do not rule out that the impaired expression of these genes might be a late event in the clinical progression of the disease, since, most AD patients showed high Braak and Thal scores.

Protein levels of these immune factors in AD brains were not measured, since many variables, such as post-mortem latency, disease stage or duration, may increase case protein variability, and this is a limitation of our investigation. Moreover, astrocyte or microglia classical neuropathological markers of activation were not investigated. Therefore, the contribution of these cell populations to brain mRNA levels of IRF7, MED23, IL28B, and IFN-α remains to be explored. However, astrogliosis, along with a decrease of neuron number, are classical neuropathology hallmarks of the AD brain [42] and increased or decreased mRNA levels might also be related to the brain area balance of these different cell populations.

Our data showed that different factors, such as brain area locations and individual genetic makeup, along with other undetermined factors, affect gene expression of innate immunity components and indirectly support the notion that impaired innate immune responses against brain insults, such as viruses, bacteria, fungi or their products, might accelerate neurodegenerative mechanisms in the elderly. A recent book elaborates in detail the role of infective agents in AD [43], and the present investigation agrees with the notion of infection association with AD.

In conclusion, the maintenance of efficient immune responses during aging might slow down neurodegenerative mechanisms associated with senile dementia and affect both the prevalence and incidence of AD. Further investigations regarding infections, immune defensive mechanisms, and AD progression might open new ways for AD prevention and therapy.

## 4. Materials and Methods 

### 4.1. Post-Mortem Human Brain Tissue

Post-mortem brain tissues of AD patients and age-matched nondemented controls (ctrl) were obtained from the Netherlands Brain Bank (NBB; Amsterdam, The Netherlands), in accordance with rules and regulations of the Ethical Code from BrainNet Europe. Patients or their next of kin gave written informed consent to NBB for brain autopsy and use of tissue and clinical information for research purposes. This study was approved by the NBB scientific committee and conditions for transferring and using brain samples regulated by the Material Transfer Agreement (MTA). Neuropathological evaluation and AD pathology staging followed Braak and Braak criteria for neurofibrillary tangles (NFTs) and Thal criteria for amyloid deposition [44,45].

AD brain tissues were stored in liquid nitrogen, and 20 μm thick slices of the hippocampus and temporal cortex were cut at −20 °C, collected in RNAse free Eppendorf vials, and stored at −80 °C for the following molecular analyses.

Twenty-nine AD hippocampus brain samples, nineteen AD temporal cortex brain samples, six hippocampus, and four temporal cortex samples from ctrl cases were included in this study. The final selection was based on the availability of DNA and RNA samples of good quantity and quality.

### 4.2. Genomic DNA Isolation

Genomic DNA was obtained from frozen samples and purified according to the Phenol:Chloroform:Isoamyl Alcohol (25:24:1) extraction’s protocol (Sigma–Aldrich, St. Louis, MO, USA) after overnight incubation with proteinase K 10 mg/mL (Roche, Basel, Switzerland) and ATL buffer (Qiagen, Hilden, Germany). Absorbance measurements were made on a NanoDrop 1000 (Thermo Scientific, Wilmington, DE, USA), and the ratio of absorbance at 260 nm and 280 nm was used to assess the purity of DNA samples, further stored at −80 °C.

### 4.3. RNA Isolation

RNA extraction from frozen hemi-brain hippocampus or temporal cortex samples was performed using an RNA-Bee kit (AMSBIO, Cambridge, MA, USA) according to the manufacturer’s instructions. Total RNA was purified according to phenol-chloroform standard extraction after overnight incubation with proteinase K. Absorbance measurements were made on a NanoDrop 1000 (Thermo Scientific, Wilmington, DE, USA), and the ratio of absorbance at 260 nm and 280 nm was used to assess the purity of RNA samples, further stored at −80 °C.

### 4.4. Quantitative Reverse-Transcription Polymerase Chain Reaction

RNA (50 ng) was converted to cDNA retro transcribed using the iScript™ cDNA Synthesis Kit (Bio-Rad, Hercules, CA, USA) following the manufacturer’s instructions. PrimerPCR™ assay for real-time PCR gene expression analysis was performed using SsoAdvanced™ Universal SYBR^®^ Green Supermix (Bio-Rad, Hercules, CA, USA) according to the manufacturer’s instructions. Bio-Rad pre-validated primer pairs for target genes IRF7 (qHsaCED0007783), MED23 (qHsaCID0007348), IL28B (qHsaCED0038284), IFN-α (qHsaCED0037471), and for two reference genes were used. CYC1 (qHsaCED0047348) and EIF4A2 (qHsaCED0023870) were selected as reference genes for normalization, as suggested by Penna and colleagues [46].

Quantitative PCR assay (q-PCR) for gene expression analysis was realized in a CFX96 Touch™ System instrument (Bio-Rad, Hercules, CA, USA), and all reactions were run in triplicate in 96-well optical plates. q-PCR data were analyzed by Bio-Rad CFX Manager™ Software (version 3.1, Bio-Rad, Hercules, CA, USA). Using the 2−^ΔΔCt^ method [47], the gene expression data were computed as the gene expression fold change after normalization to the two reference genes (CYC1 and EIF4A2).

### 4.5. SNPs Detection

TaqMan^®^ SNP Genotyping Assay (Applied Biosystems, Foster City, CA, USA) was used for genotyping AD patients according to the manufacturer’s instructions. It included an unlabeled PCR primer pair to detect specific targeted SNP and two different Taqman^®^ probes for detecting two SNP alleles: one probe was labeled with VIC^®^ dye and the other one with 6-FAM^®^ dye. Allelic discrimination was performed by probe signal intensity from PCR (RT-PCR) using a CFX96 Touch™ System instrument (Bio-Rad, Hercules, CA, USA).

APOE ε allele (rs429358 and rs7412) was assessed by RT-PCR using Taqman^®^ probes according to the manufacturer’s instructions. The upstream variant of IRF7 (rs6598008 A/G), MED23 (rs3756784 T/G), and IL28B (rs12979860 C/T) genes were also analyzed by RT-PCR using Taqman^®^ probes according to the manufacturer’s instructions.

### 4.6. Statistical Analysis

Statistical analysis was performed using the Statistical Package for the Social Sciences (version 22.0; SPSS Inc, Chicago, IL, USA) and two-sided *p*-values are presented.

After careful quality control of the normalized data, a generalized linear model analysis (ANOVA) followed by Bonferroni post-test or unpaired *t*-test was used to analyzed differences in gene expression data between groups.

## 5. Conclusions

Our data indirectly support the notion that impaired innate immune responses against brain insults induced by microorganism infection might accelerate neurodegenerative mechanisms in the elderly.

Maintenance of efficient immune responses in the elderly might slow down neurodegenerative mechanisms associated with age-related cognitive decline and affect the prevalence and incidence of AD.

## Figures and Tables

**Table 1:** Demographic and clinical features of demented patients.

ID	Age (Years)	Gender	Clinical Diagnosis	DD	PMI (h)	BW (gr)	Cause of Death	Brain Area
1	78	F	MID	7	4.35	1043	Pneumonia, cachexia, decubitus	Hip; Temp
2	78	F	AD	10	4.50	1105	Gastrointestinal bleeding	Hip; Temp
3	81	M	AD	5	4.50	1253	Probable CVA and sepsis with unknown underlying disease	Hip
4	95	F	MID possibly mixed with AD	7	3.40	1032	Aspiration pneumonia	Hip
5	86	M	MID	6	3.40	1206	Metabolic disturbances, dehydration, metastasized prostate carcinoma	Hip; Temp
6	84	F	MID	na	3.40	1109	Dehydration, cachexia	Hip; Temp
7	92	F	VD	3	3.40	1115	Bleeding gastric ulcer	Hip
8	73	F	AD	12	3.40	1082	Sepsis, cachexia	Hip
9	67	F	presenile AD	15	3.40	945	Cachexia	Hip; Temp
10	93	M	AD	1	3.40	1210	na	Hip
11	93	M	MID	9	3.40	1220	Cardiac failure caused by pneumonia	Hip
12	69	M	na	na	3.40	1241	Dehydration, cachexia	Hip
13	89	F	AD	11	3.40	1211	na	Hip
14	84	F	AD	na	3.40	1098	Dehydration	Hip
15	91	F	AD	5	3.40	1101	Dehydration, cachexia, and probable CVA	Hip; Temp
16	93	F	AD	4	2.30	1045	Cachexia	Hip; Temp
17	86	F	AD	10	5.05	998	Uncontrolled anti-coagulation in combination with general deterioration after hip prosthesis	Hip
18	94	F	VD after CVA	6	5.05	1170	Cachexia and dehydration, decubitus	Hip
19	75	F	AD	9	6.00	1129	Dehydration	Hip
20	89	F	VD	19	4.30	1185	Pneumonia	Hip; Temp
21	74	M	AD	2	5.35	1380	Anemia, hepatic dysfunction, extensive lymphadenopathy by M. Kimura	Hip
22	75	F	AD	6	15.0	1230	Cardiac arrest	Hip
23	94	F	AD	6	8.05	1053	Cachexia	Hip
24	90	F	AD	6	5.40	1100	CVA, advanced AD	Hip
25	88	F	na	1	6.45	1170	Natural death	Hip
26	66	F	AD	6	6.30	1190	Acute heart failure by advanced dementia syndrome	Hip
27	82	F	VD	6	5.55	1225	Cardiac arrest with dehydration after MI	Hip
28	99	F	AD	5	3.30	1150	Airway infection	Hip
29	60	M	AD possibly of Lewy Body type	3	6.15	1241	Cachexia and dehydration by dementia syndrome	Hip; Temp
30	81	F	AD	2	6.10	1295	Cachexia and dehydration by dementia syndrome	Temp
31	57	M	presenile AD	6	3.50	1055	Aspiration pneumonia	Temp
32	69	M	VD, CI	3	7.10	1173	Aspiration pneumonia	Temp
33	86	M	AD	6	6.15	1331	Possible cardiac arrest after gastroenteritis by AD	Temp
34	92	F	AD	5	3.25	1105	Probable CVA	Temp
35	88	F	AD	12	12.15	935	Cachexia and decubitus	Temp
36	85	F	MID	5	4.45	1310	Shock due to acute abdomen	Temp
37	94	F	na	na	5.00	1410	Double side pneumonia	Temp
38	80	M	na	na	4.00	1328	Unknown	Temp
39	85	M	AD possibly in combination with MID	5	4.25	1458	Sudden death (suspected heart-failure)	Temp

Abbreviations: DD, Disease Duration; PMI, Post-mortem Interval; BW, Brain Weight; F, female; M, male; MID, Multi-Infarct Dementia; AD, Alzheimer’s Disease; VD, Vascular Dementia; CI, Cognitive Impairment; CVA, Cerebrovascular Accident; MI, Myocardial Infarction; Hip, hippocampus; Temp, mid temporal cortex; na, not applicable.

**Table 2:** Alzheimer’s disease (AD) neuropathological features.

ID	Pathological Diagnosis	Grade (Braak, NTF)	Grade (Thal, Aβ)
1	AD	5	C
2	AD	5	C
3	AD	4	C
4	AD; meningioma	4	C
5	AD	3	A/B
6	AD	5	C
7	AD; infarction	4	C
8	AD	5	C
9	AD	6	C
10	AD	5	C
11	AD; caa; arg	4	C
12	AD	6	C
13	AD	5	C
14	AD; infarction	5	C
15	AD	4	C
16	AD	4	C
17	AD	4	C
18	AD; infarction	4	C
19	AD; caa	5	C
20	AD	6	C
21	AD	6	C
22	AD	5	C
23	AD	4	C
24	AD; infarction	6	C
25	AD	5	C
26	AD	5	C
27	AD	4	C
28	AD	4	C
29	AD	6	C
30	AD	5	C
31	AD	6	C
32	AD; infarction	5	B
33	AD	5	C
34	AD; ischemia	5	C
35	AD	4	C
36	AD	5	C
37	AD	4	C
38	AD	4	C
39	AD	5	B/C
			

Abbreviations: caa = congophilic angiopathy; arg = argyriphilic grain disease.

## Abbreviations

AD	Alzheimer’s Disease
IRF	Interferon Regulatory Factor
MED23	Mediator Complex 23
IL	Interleukin
IFN	Interferon
APOE	Apolipoprotein
Aβ	Amyloid-β
AMPs	Antimicrobial Peptides
HSV-1	Herpes Simplex Virus 1
HHV	Human Herpes Virus
SNP	Single Nucleotide Polymorphism
EBV	Epstein-Barr Virus
DD	Disease Duration
PMI	Post-Mortem Interval
CXCL13	Chemokine (C-X-C motif) Ligand 13
NBB	Netherlands Brain Bank
MTA	Material Transfer Agreement
NTFs	NeuroFibrillary Tangles
CYC1	Cytochrome C1
EIF4A2	Eukaryotic Initiation Factor 4A2
q-PCR	Quantitative PCR assay
